# Hollywood 3D: What are the Best 3D Features for Action Recognition?

**DOI:** 10.1007/s11263-016-0917-2

**Published:** 2016-06-21

**Authors:** Simon Hadfield, Karel Lebeda, Richard Bowden

**Affiliations:** CVSSP, University of Surrey, GU2 7XH Guildford, UK

**Keywords:** Action recognition, In the wild, 3D, Structure, Depth, 3D motion, Hollywood 3D, Benchmark

## Abstract

Action recognition “in the wild” is extremely challenging, particularly when complex 3D actions are projected down to the image plane, losing a great deal of information. The recent growth of 3D data in broadcast content and commercial depth sensors, makes it possible to overcome this. However, there is little work examining the best way to exploit this new modality. In this paper we introduce the Hollywood 3D benchmark, which is the first dataset containing “in the wild” action footage including 3D data. This dataset consists of 650 stereo video clips across 14 action classes, taken from Hollywood movies. We provide stereo calibrations and depth reconstructions for each clip. We also provide an action recognition pipeline, and propose a number of specialised depth-aware techniques including five interest point detectors and three feature descriptors. Extensive tests allow evaluation of different appearance and depth encoding schemes. Our novel techniques exploiting this depth allow us to reach performance levels more than triple those of the best baseline algorithm using only appearance information. The benchmark data, code and calibrations are all made available to the community.

## Introduction

Recognising actions “in the wild” is useful for many applications including surveillance, automatic video indexing/search and assisted living. Huge intra-class variation is inherent to recognition in the wild, caused by the wide variety of environments, actors, viewpoints and action styles. We address this issue by exploiting the invariances inherent in 3D data, and proposing new approaches to using and encoding this information, to provide better generalisation capability.

There has been extensive previous work on “in the wild” action recognition in 2D data. Likewise, there has been significant work in recent years, on action recognition for 3D data “in the lab”, due to the introduction of cheap consumer depth sensors. However, the crossover between the two areas, 3D recognition in the wild, has rarely been considered. It is important to address this problem as new sources of 3D data such as mobile devices are emerging, in addition to increasing levels of 3D broadcast data from television networks & film studios.

In the past, benchmark datasets such as KTH (Schuldt et al. 2004), Weizmann (Blank et al. 2005) or Kinect based datasets (Li et al. 2010; Cheng et al. 2012) have been invaluable in providing comparative benchmarks to examine how competing approaches perform in action recognition and detection. However, these staged datasets now routinely have reported performance rates over 90 %, suggesting that they are reaching the end of their service to the community. “In the wild” datasets such as Hollywood (Laptev et al. 2008), Hollywood2 (Marszalek et al. 2009) and our Hollywood 3D (Hadfield and Bowden 2013) provide a more challenging problem due to huge variability in appearance. These more natural datasets consist of actions extracted from a variety of Hollywood feature films. They provide a new level of complexity to the recognition community, arising from the natural within-class variation of unconstrained data, including unknown camera motion, viewpoint, lighting, background and actors, and variations in action scale, duration, style and number of participants. While this natural variability is one of the strengths of the data, the lack of structure or constraints make classification an extremely challenging task.

The use of depth information can help to mitigate some of these factors. Lighting variations are generally not expressed in depth data, and actor appearance differences are eliminated (although differences in body shape remain). Additionally, depth provides useful cues for background segmentation, and occlusion detection. However, this also introduces new problems such as the inconsistency of 3D data obtained from disparate sources with unknown calibrations.

**Fig. 1:**
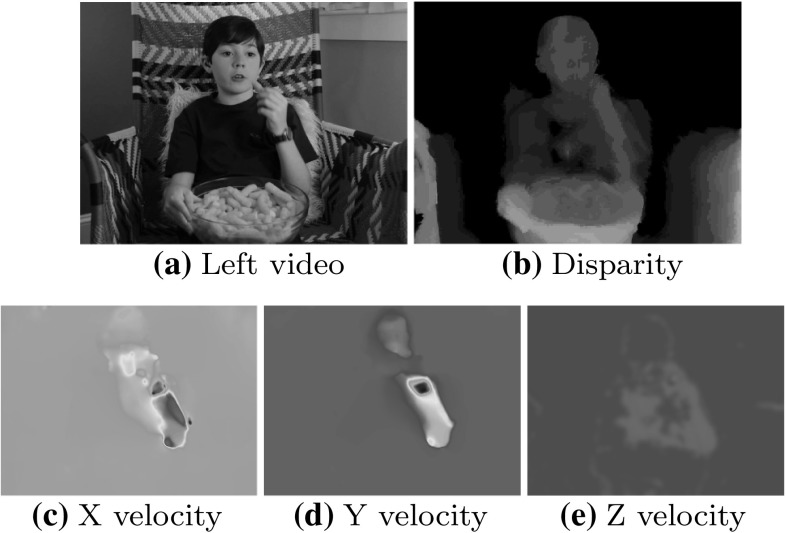
The appearance and disparity (*top row*) for a *Eat* action from the Hollywood 3D dataset. The 3D motion field is also shown on the *bottom row*. The primary motion is concentrated on the arm and head, which move towards each other

**Fig. 2:**
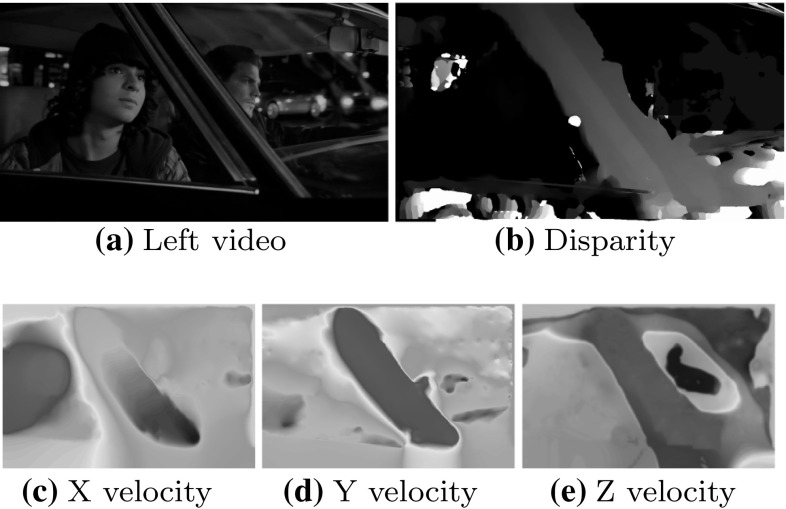
The appearance and disparity (*top row*) for a *Drive* action from the Hollywood 3D dataset. The 3D motion field is also shown on the *bottom row*. Note that the primary motion occurs on the foreground regions of the car, with secondary x and y motion on the passengers

This paper discusses the Hollywood 3D benchmark dataset for 3D action recognition in the wild. In addition a broad experimental baseline is produced; many techniques for 2D “in the wild” recognition are extended to operate on depth data, including 2 feature descriptors and 5 interest point detectors. The effect of incorporating this depth data is comprehensively examined and the full source code of all these baseline techniques is provided to stimulate further research. Another novel feature descriptor is also proposed based on recent advances in the estimation of 3D motion fields (shown in Figs. 1, 2) (Hadfield and Bowden 2014b). This is coupled with a robust stereo auto-calibration framework to remove calibration inconsistencies from the resulting features without human intervention. The resulting calibrations are also provided to accompany the dataset. Finally a novel viewpoint-invariant feature encoding scheme is proposed to make it easier to recognise the similarities between different shots of the same action.

Compared to the initial presentation of the Hollywood 3D benchmark (Hadfield and Bowden 2013) and subsequent extensions (Hadfield et al. 2014), in addition to unifying the work in a single manuscript, this paper includes three primary contributions. Firstly the evaluation has been significantly expanded including the popular dense trajectories (Wang et al. 2011) encoding technique. Secondly a comparison of techniques other authors have proposed for this benchmark has been added, including deep-learning based approaches (Iosifidis et al. 2014b, a), implicit calibration (Konda and Memisevic 2013) and new feature descriptors (Mademlis et al. 2014; Konda and Memisevic 2013). This helps to provide additional insight into the field, beyond what was possible with the initial baseline experiments. Finally, deeper discussion is also included of the scene-flow encoding, compared to the initial work in Hadfield et al. (2014). This includes a discussion of the 3D motion estimation pipeline.

## Related Work

A common practice in action recognition is to focus attention on parts of the scene which are identified as salient by interest point detectors (Laptev and Lindeberg 2003). One advantage is tractability, reducing the quantity of data for subsequent processing (Gilbert et al. 2011; Laptev et al. 2008; Li et al. 2010). In the past, this approach has also helped to suppress irrelevant background information. Note that in this paper we make a distinction between saliency and interest points. The estimated “interest” of any image point is a continuous number referred to as the point’s *saliency*. The parts of an image which have a saliency score greater than a particular threshold are then collectively referred to as the set of *interest points*. Generally speaking interest points are also filtered via non-maxima suppression, to prevent duplicate entries.

Some approaches segment the actor, for example using the Kinect’s user mask (Gorelick et al. 2007; Li et al. 2010; Cheng et al. 2012). This enables complex “volumetric” descriptions of the actor’s body over time (Yang et al. 2012; Wang et al. 2012; Vieira et al. 2012; Oreifej and Liu 2013). However, for “in the wild” action recognition it remains challenging to segment the actor reliably, due to noisy 3D data, cluttered environments, and scenes containing multiple people. This is still an active area of research, with current techniques (Desai and Ramanan 2012; Yao and Fei-Fei 2012; Hoai et al. 2014) only able to provide rough bounding boxes, rather than the per pixel segmentations generally used in volumetric description. As such, it is still common to use interest point detectors for recognition in the wild. Recently an intermediate encoding approach, called Dense Trajectories, has been successful (Wang et al. 2011; Gilbert and Bowden 2014). As the name implies, this is based on densely sampled local features, with temporal accumulation along the trajectories. In this paper we explore both interest point and dense trajectory encoding schemes.

When the salient parts of the sequence have been detected, it is common to compute local descriptors of these spatio-temporal region. Some of the most popular descriptors have been based on the gradient of the appearance information (Schuldt et al. 2004; Laptev and Perez 2007), spatio-temporal extensions to SIFT and SURF descriptors (Scovanner et al. 2007; Willems et al. 2008) and 2D motion information (Dalal et al. 2006; Messing et al. 2009). However, there has been little previous work on feature descriptors including depth information (which is generally encoded directly at the holistic level, with the aid of user masks).

**Fig. 3:**
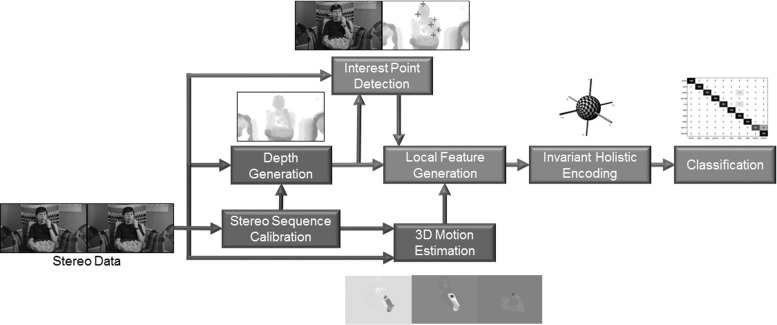
“In the wild” action recognition pipeline, making use of depth information at various stages. The *green* elements refer to dataset pre-processing, while *blue* elements relate to the recognition pipeline (Color figure online)

The local descriptors from salient regions can then be accumulated into a single holistic description of the sequence. One way to achieve this is to take inspiration from highly successful Bag-of-Words techniques in the field of object recognition, and include an additional temporal dimension. This entails creating a codebook of exemplar features, and then accumulating occurrences of these exemplars spatially and temporally across the sequence. This is invaluable for generalisation, as it provides invariance to a range of important deformations, such as spatial and temporal translation, stretching and reflection. However, the accumulation also leads to much of the relational information being discarded, such as the spatial configuration and temporal ordering of features. Laptev *et al*.attempt to mitigate this by splitting the spatio-temporal volume into sub-blocks, creating a descriptor for each sub-block, and concatenating them to create the sequence descriptor (Laptev et al. 2008). Sapienza *et al*.follow a similar vein, encoding individual sub-sequences, however rather than concatenating to create a single descriptor, they employ Multiple Instance Learning (MIL) (Sapienza et al. 2012). This accounts for some parts of the sequence being irrelevant, for example before and after the action. In this paper we propose a number of novel encoding schemes specific to our 3D motion features, which incorporate additional invariances such as scale and viewpoint invariance.

Some approaches avoid the bag-of-words holistic description route. These include data-mining and voting schemes (Gilbert et al. 2011) and chains of single-frame recognitions (for example using HMMs Brand et al. 1997). The lack of the accumulation step makes the learning task more complex due to the lack of invariances. However, it has the advantage that the exact location and time of the action is estimated. There has also been a recent rise in techniques based on deep learning, where bag-of-words is obviated. These range from simply pre-processing the input images (Simonyan and Zisserman 2014; Karpathy et al. 2014) to convolving over time (Ji et al. 2013) and estimating motion patterns (Simonyan and Zisserman 2014).

## Paper Overview

The structure of the paper roughly mirrors the flow of the approach, as shown in Fig. 3 (green elements indicate dataset pre-processing steps, and blue elements relate to the recognition pipeline). First, Sect. 4 covers the stereo data extraction approach, with details of the Hollywood 3D dataset. The proposed auto-calibration technique is then described in Sect. 5 and the results are used to extract 3D structure and motion information from the dataset as described in Sect. 6 using scene flow estimation.

The recognition pipeline is performed in 3 stages. Firstly salient points are detected using a range of detection schemes including a number of new schemes which incorporate the depth information, as discussed in Sect. 7. Next, feature descriptors are extracted from these salient points, encoding both appearance and depth information. These are discussed in detail in Sect. 8, and include extensions of two well known techniques and a novel motion descriptor based on 3D motion fields. These local descriptors are then accumulated over the sequence, with 3D motion features using viewpoint invariant encoding. In Sect. 9 we present results classifying these holistic descriptors using a Support Vector Machine (SVM), while exploiting depth data at various stages of the pipeline. Our conclusions about the benefits of depth data in natural action tasks, and the relative merits of the presented approaches, are then summarised in Sect. 10 where the use of depth consistently outperforms appearance-only recognition.

**Table 1:** The number of unique training and test sequences for each action in the dataset

*NoAction*	*Run*	*Punch*	*Kick*	*Shoot*	*Eat*	*Drive*	*UsePhone*	*Kiss*	*Hug*	*StandUp*	*SitDown*	*Swim*	*Dance*	Total
44	38	10	11	47	11	51	21	20	9	22	14	16	45	359
34	39	9	11	50	11	47	20	20	8	21	13	17	7	307

The top row is the training set and the bottom row is the test set

## Extracting 3D Action Clips from Movies

There has been a sharp rise in commercially available 3D content, due to the emergence of high definition home media such as BluRay™and the introduction of 3D displays into the consumer market. Unfortunately much of this data is not suitable for action recognition. Most of the earlier 3D films were constructed via post-processing techniques (i.e. rotoscoping) from the original 2D data, and is fundamentally artificial, created for effect only. When depth data is extracted from these films, any fine-detailed depth variations within objects are missing, with scene depth simplified into a number of discrete depth planes. Additionally, films generated entirely through CGI (where 3D versions are much easier to produce), are unlikely to provide transferable information on human actions. For this dataset, we have focused on content captured using commercial camera rigs such as James Cameron’s Fusion Camera System™or products from 3ality Technica. These use real stereo cameras rigs, making it possible to reconstruct accurate 3D depth maps.

Most 3D films are too recent to have publicly available transcriptions, and subtitles alone rarely offer action cues, so automatic extraction techniques such as those employed by Marszalek et al. (2009) are currently not feasible and manual labelling was used. This also ensures that all examples are well segmented from their carrier movies. This approach led to 650 clips (after class balancing) spread across 13 action classes, and a collection of 78 sequences containing no actions. These *NoAction* clips were automatically extracted as negative data, while ensuring no overlap with positive classes. The dataset was extracted from 14 films^1^ and is publicly available (Hadfield and Bowden 2013). In total the dataset contains over an hour of footage.

Within the dataset, actions are temporally localized to the frame level, ensuring non-discriminative frames at the start and end of sequences do not confuse training, and also improving separation of the *NoAction* class. The data is high definition (1920 by 1080 resolution) and is provided for both the left and right viewpoints at 24 frames per second.

To emphasize generalization, the 14 films comprising the dataset were split between the train and test sets on a per action basis. As such, each action is tested on actors and settings not seen in the training data. Certain actions are more common than others, and as in the Hollywood and Hollywood2 datasets, this prior distribution is reflected in the dataset. The number of training and test clips is shown in Table 1.

## Stereo Sequence Auto-calibration

Every sequence in the dataset comprises an appearance from both the left and right viewpoint (extracted from the original film). For the stereo calibration of these viewpoints (i.e. the rotation and translation between the views and the intrinsics parameters of both cameras), the initial release of Hollywood 3D included only a single generic calibration for the entire dataset. This was based on the types of cameras and lenses commonly used in broadcast footage. However, this is overly simple as in reality the calibration varies greatly between films (where different cameras may be used), and even between different shots of the same film (the stereo-rig may be modified to change the strength of the perceived depth). This makes extracting consistent 3D information difficult from sequence to sequence, introducing a huge amount of artificial variation to the action classes and making recognition even more challenging. To mitigate this issue, we employ a stereo auto-calibration stage operating on the video pairs, which ensures extracted 3D features are more comparable across sequences.

The first step towards calibrating the pair of video sequences $I^{l}$ and $I^{r}$, each of which consists of $n$ frames (${{\varvec{I}}^{l}_{1...n}}$ and ${{\varvec{I}}^{r}_{1...n}}$), is to detect a set of candidate correspondences. In this paper, sets ($S^{l}$ and $S^{r}$) of SIFT (Lowe 2004) points are extracted, where

1 $$\begin{aligned} S^{}=\left\{ {\varvec{s}}^{}_{} : \text {DoG} \left( {{\varvec{I}}^{}_{\tau }} \left( x,y\right) \right) > \lambda _{s}\right\} , \end{aligned}$$

based on the threshold $\lambda _{s}$. Non-maxima suppression is also applied to avoid prevent multiple interest points being generated by the same feature. Each resulting SIFT point comprises a space-time location ${\varvec{s}}^{}_{i}=(x,y,\tau )$ (where $x,y$ is the image position and $\tau$ is the frame number). Additionally each point has an associated SIFT descriptor ${\varvec{f}}^{}_{i}$. Correspondences between point detections are calculated subject to the condition that their descriptors are closer than a threshold $\lambda _{f}$, and that they occur at the same frame in both sequences,

2 $$\begin{aligned} C = \left\{ \left( {\varvec{s}}^{l}_{i},{\varvec{s}}^{r}_{j}\right) : |{\varvec{f}}^{l}_{i}-{\varvec{f}}^{r}_{j}|< \lambda _{f} \text { and }\tau ^{l}_{i}=\tau ^{r}_{j} \right\} . \end{aligned}$$

Given this set of cross sequence correspondences, the epipolar geometry of the scene is estimated using 7-point RANSAC with Local Optimisation (Lebeda et al. 2012). The fundamental matrix is estimated by

3 $$\begin{aligned} {\varvec{F}}=\mathop {{{\mathrm{arg\,min}}}}\limits _{ {\varvec{F}'} } \sum {\epsilon _{s} \left( {{\varvec{s}}^{l}_{i}},{\varvec{s}}^{r}_{i}| {\varvec{F}'} \right) }, \end{aligned}$$

where $\epsilon _{s}$ is the Sampson error (linearised approximation to projection error). In this work $\epsilon _{s}$ also applies a truncated quadratic cost function (as in MSAC), which provides an approximation to the maximum likelihood estimate (Torr and Zisserman 1998).

Given the estimated ${\varvec{F}}$ we can also extract the set of inlier correspondences,

4 $$\begin{aligned} \hat{C}= \left\{ \left( {\varvec{s}}^{l}_{i},{\varvec{s}}^{r}_{i}\right) : \left| {{\varvec{s}}^{l\top }_{i}}{\varvec{F}}{\varvec{s}}^{r}_{i}\right| < \lambda _{r} \right\} , \end{aligned}$$

which obey the epipolar constraints estimated. For the experiments in this paper, the detection, matching and inlier thresholds ($\lambda _{s}$, $\lambda _{f}$ and $\lambda _{r}$ respectively) use the default values suggested by their respective authors.

### Full 3D Sequence Calibration

Estimating the epipolar geometry between the sequences is only the first step to consistent 3D calibration. Next the focal length (and hence the Essential matrix ${\varvec{E}}$) must be estimated. This is feasible, subject to the assumption of square pixels, and that focal length is consistent between the two sequences (this assumption is reasonable, as stereo capture rigs generally utilise the same type of camera for both views, and it would be jarring for the audience if one eye was zoomed differently to the other). This can then be combined with constraints on the rank of ${\varvec{F}}$, and the trace of ${\varvec{E}}$, to construct a Polynomial Eigenvalue Problem (PEP) which may be efficiently solved (Kukelova et al. 2008). As with the estimation of ${\varvec{F}}$, this is solved in a RANSAC framework, using the inliers to the epipolar geometry $\hat{C}$.

**Fig. 4:**
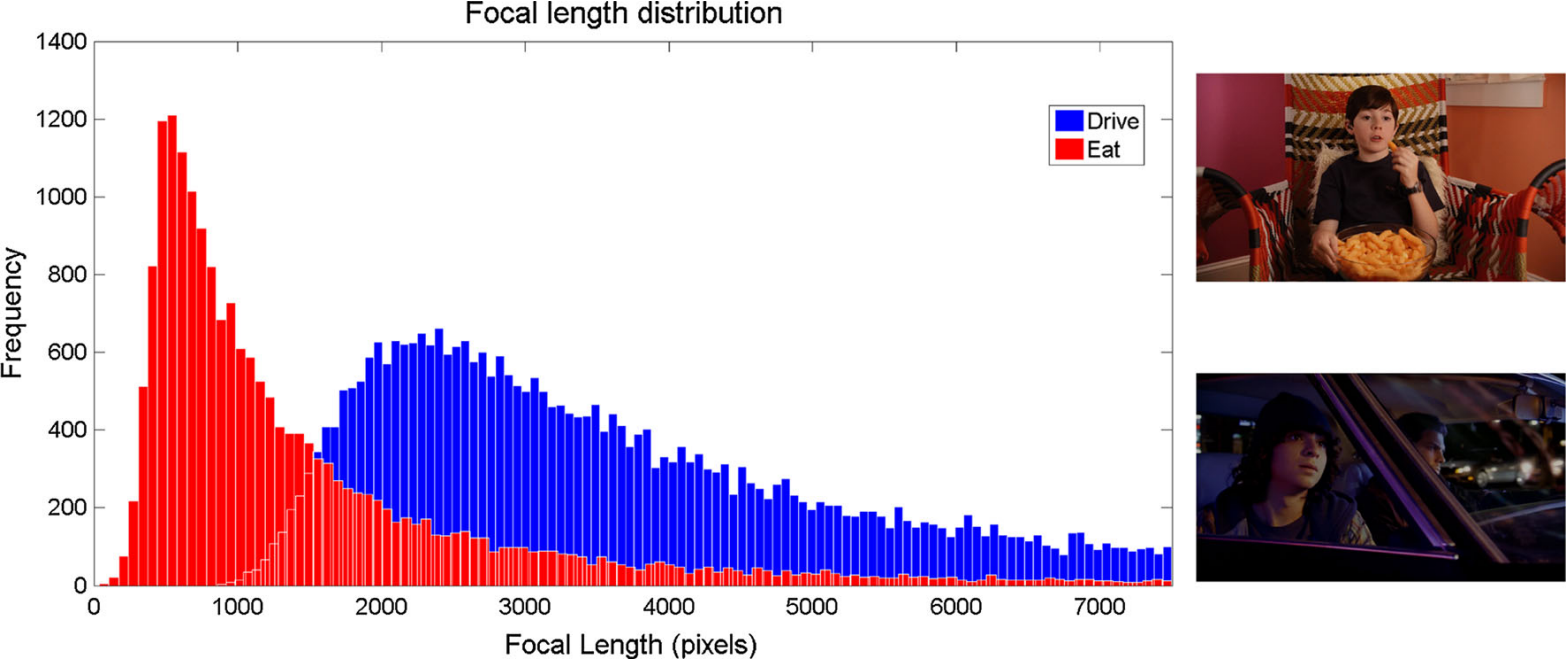
Distribution of estimated focal lengths over 20,000 repetitions, on the 2 different sequences pairs shown (wide-angle, close-up *Eat* shot, and extreme zoom *Drive* shot)

Unfortunately, for 3D footage it is very common for cameras to be in a near-parallel configuration. This adversely affects the stability of the PEP, which (although deterministic) may become sensitive to changes in the input correspondences $\hat{C}$. In other words, for a given $\hat{C}$ a particular ${\varvec{E}}$ is estimated consistently. However, adding or removing a small number of points from $\hat{C}$ can in some cases lead to significant differences in the estimated ${\varvec{E}}$. Due to the offline nature of the auto-calibration system, coupled with efficient PEP solvers, the process can be repeated a number of times. Each iteration finds a slightly different ${\varvec{F}}$ and $\hat{C}$ which in turn leads to a different ${\varvec{E}}$.

Figure 4 shows the distribution of focal lengths estimated over a large number of repetitions, for two sequence pairs with different levels of zoom. The distribution of focal lengths arising due to the near-parallel camera configuration follows the log-normal distribution which should be expected from a multiplicative entity such as the focal length. As such, we can achieve robustness to near-parallel cameras, by taking the mode of this distribution, for each sequence pair. In our experiments we use 100 calibration repetitions to model this distribution, which takes a few minutes in our single thread Matlab implementation. This is reasonable as it only needs to be performed once per sequence and is part of the pre-processing (i.e. it does not need to be repeated for each new experiment).

Finally, given our robust estimate of ${\varvec{E}}$, it is possible to estimate the projection matrices ${\varvec{P}}^{l}$ and ${\varvec{P}}^{r}$ for the cameras (Hartley and Zisserman 2000). This leads to 4 possible solutions. We select the solution that maximizes the number of corresponding point pairs $\hat{C}$ intersecting in front of the cameras,

5 $$\begin{aligned} {\varvec{P}}^{l},{\varvec{P}}^{r} = \mathop {{{\mathrm{arg\,max}}}}\limits _{{\varvec{P}}^{'l},{\varvec{P}}^{'r}} \sum _{({\varvec{s}}^{l}_{i},{\varvec{s}}^{r}_{i}) \in \hat{C}}{\text {sign}(d_{l}) + \text {sign}(d_{r})} , \end{aligned}$$

where $d_{l}$ and $d_{r}$ are the distances along the rays defined by homogeneous points $\bar{{\varvec{s}}}^{l}_{i}$, $\bar{{\varvec{s}}}^{r}_{i}$ and ${\mathcal D}$ is the 3D position of the rays intersection,

6 $$\begin{aligned} d_{l}\bar{{\varvec{s}}}^{l}_{i} = {\varvec{P}}^{'l} {\mathcal D} \;\text { and }\; d_{r}\bar{{\varvec{s}}}^{r}_{i} = {\varvec{P}}^{'r} {\mathcal D} . \end{aligned}$$

**Fig. 5:**
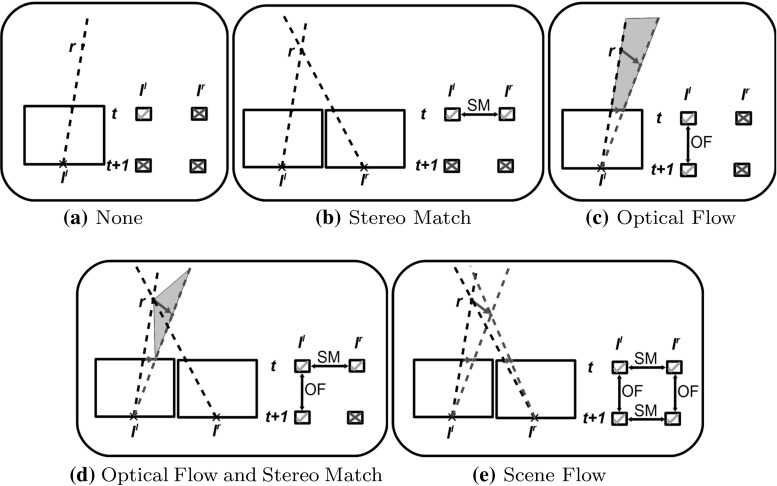
Detection of correspondences between the two cameras, and between two points in time (shown in *black* and *green*). This illustrates the scene flow estimation task, and it’s relation to optical flow (OF) and stereo matching (SM) (Color figure online)

The proposed approach to stereo sequence calibration has some limitations. Firstly, lens distortion is not included in the model. This is acceptable for a wide range of footage from Kinect devices and broadcast sources, which generally exhibit little distortion, however this may be an issue for upcoming 3D mobile devices. Secondly, in order to exploit correspondences over entire sequences, a consistent focal length is assumed (i.e. no zooming within a single shot). In theory the technique could be extended by collecting correspondences within a sliding window, and estimating a time varying focal length. However, to obtain a sufficient number of correspondences within the window, it becomes necessary to reduce robustness by allowing weaker matches. In practice the “no zooming” assumption is reasonable as most modern broadcast footage prefers to zoom between shots rather than within shots. Additionally, zooming is particularly unlikely while important actions are being performed. As a result only around 1 % of the sequences in the Hollywood 3D dataset contain a zoom. Hence, the robustness gained by utilising this assumption is far more significant than any limitations it imposes.

The final limitation is that the reconstructions obtained by our calibration technique (available online), are only consistent with each other up to a similarity transform (for comparison, reconstructions using the original generic calibration was consistent up to a homography). The removal of projective distortions does reduce the variability in the data, but the remaining scale ambiguity still must be addressed during encoding.

## Structure and 3D Motion Estimation

Once we have an estimated calibration, we can extract 3D structure and motion information from the dataset. For the structural information (i.e. stereo matching) we use a GPU-accelerated bilateral grid filtering approach, as described by Richardt et al. (2010). This technique attempts to estimate smooth but edge-preserving scene structures based on filtering theory, and unlike many other modern stereo techniques, scales well to the large amount of high-resolution content in the dataset.

To replace the 2D optical flow descriptor with a 3D “scene flow” descriptor, the 3D motion field was estimated. At time $t$ this motion field is related to 4 different input frames. As shown in Fig. 5 these are ${{\varvec{I}}^{l}_{t}}$, ${{\varvec{I}}^{r}_{t}}$, ${{\varvec{I}}^{l}_{t+1}}$ and ${{\varvec{I}}^{r}_{t+1}}$, and scene-flow estimation can be seen as trying to find the scene structure at 2 different times, while also estimating exactly how one structure warps into the other. As such, the task is then to find a set of 6D vectors ${\varvec{w}}$ (comprising 3D world position $x,y,z$ and 3D world velocity $\dot{x},\dot{y},\dot{z}$), which are consistent with all 4 images observations. There are many approaches to achieve this, but for estimating 3D motion fields for action recognition, we use the sampling based Scene Particles (Hadfield and Bowden 2014a) approach. This is well suited to action recognition as it provides excellent performance at object boundaries (Hadfield and Bowden 2011) (where most salient point detections occur during human action sequences). In addition the approach is orders of magnitude faster than competing variational techniques, which is vital when dealing with the large quantities of data present in action recognition datasets.

For a given ${\varvec{w}}$ we can use the estimated calibrations to find its projection in all 4 relevant images. We can then measure the quality of this ${\varvec{w}}$ based on the Brightness Constancy Assumption which is common in motion estimation algorithms (i.e. for a real point on the scene structure, its appearance should not change over time, or with viewpoint). We measure conformance to this assumption using the variance of the appearance of the 4 projected points,7where $\bar{I}$ is the mean observed appearance across all 4 frames, and ${\varvec{w}} _{\tau }$ is the 3D end point of the flow corresponding to frame $\tau$. The ${\varGamma }$ is an Intelligent Cost Function (ICF) (Hadfield and Bowden 2014b) which is a type of robust scoring function using Gaussian Processes to reflect the distribution of motion errors in real scenarios.

This ICF is trained using the Middlebury stereo dataset (Scharstein and Szeliski 2003). Real correspondences are extracted using the ground truth, alongside deliberate “erroneous matches”. These are then used to train a matching function which ideally separates correct and incorrect correspondences, while accounting for realistic lightning effects such as specularities and under/over-exposure. Conditioning the error on the observations in this way greatly improves robustness when compared to simply penalising the squared error.

We can embed this likelihood function in an efficient estimation scheme, such as a particle filter, in order to estimate consistent sets of motions ${\varvec{w}}$ over the sequence, which maximize ${\varvec{p}}({{\varvec{I}}^{l}_{}},{{\varvec{I}}^{r}_{}}\mid {\varvec{w}} )$. This efficient scheme is important, as the size of most action recognition datasets preclude the use of variational optimization based techniques (Huguet and Devernay 2007), which our experiments indicated would take thousands of years to complete.

When using particle filtering to estimate the scene flow probability distribution, special attention must be paid to the coverage of the estimate. If all the “Scene Particles” are collected within a standard approximation framework, the particles will accumulate over time. In this case they will approximate the probability distribution in confident regions of the scene (such as strong edges) with great accuracy, while areas of low accuracy such as untextured regions will be represented sparsely by a small number of samples. To resolve this issue we adopt the “ray resampling” strategy, where the population of each viewing ray is treated independently during resampling to ensure even coverage of the scene. Another way to view this procedure is that every ray is assigned its own particle filter, but with the particles being capable of moving between rays at every frame increment. For further details see Hadfield and Bowden (2014b).

## Interest Point Detection

Once the dataset has been preprocessed by extracting the structure, 3D motion and calibration information, the additional information present in this data may be exploited. This can be done at various stages of the pipeline, but we first look at interest point extraction, in order to detect more salient features, and discount irrelevant detections. The extended algorithms discussed in this section are based on the Harris Corners work by Laptev and Lindeberg (2003), the Hessian points algorithm by Willems et al. (2008) and the Separable Filters technique by Dollar et al. (2005). For a comparison of the original 2D interest point detection schemes (without the proposed depth-aware extensions), see the survey paper by Tuytelaars and Mikolajczyk (2008).

### 4D Interest Points

The Harris Corner (Harris and Stephens 1988) is a frequently used interest point detector, which was extended into the spatio-temporal domain by Laptev and Lindeberg (2003). The detector is based on the second-moment-matrix ($\mathbf{\psi }$) of the Gaussian smoothed spatio-temporal volume (*I*). Interest points are detected in the spatio-temporal volume as locations where $\mathbf{\psi }$ contains 3 large eigenvalues, i.e. there is strong intensity variation along 3 distinct spatio-temporal axes. To avoid eigenvalue calculation at every point, the following approximate formulation is used where (*u*, *v*, *w*) is a spatio-temporal position and *k* is typically 0.001:

8 $$\begin{aligned} H(u,v,w)=\text{ det }\left( \mathbf{\psi } \left( u,v,w\right) \right) -k \text{ trace }\left( \mathbf{\psi } \left( u,v,w\right) \right) ^{3}\,. \end{aligned}$$

To extend the operator into 4D, the power of the trace is increased, and $\mathbf{\psi }$ must be expanded to a 4 by 4 matrix, incorporating the differential ($I_{z}$) along *z*. However, the combination of appearance and depth streams does not constitute volumetric data (i.e. measurements are not dense along the new dimension as in an MRI scan). This is referred to as 3.5D rather than 4D data, and gradients cannot be directly calculated along the *z* axis. Instead, the relationship between the spatio-temporal gradients of the depth stream and those of the appearance stream are exploited. If $I_{x}$,$I_{y}$,$I_{t}$ are intensity gradients along the spatial and temporal dimensions and $D_{x}$,$D_{y}$,$D_{t}$ are the gradients of the depth stream (and omitting the spatio-temporal location (*u*, *v*, *w*)) a simple application of the chain rule allows us to estimate $I_{z}$.

9 $$\begin{aligned} I_{z} = \frac{~\frac{dI}{dx}~}{\frac{dz}{dx}} + \frac{~\frac{dI}{dy}~}{\frac{dz}{dy}} + \frac{~\frac{dI}{dt}~}{\frac{dz}{dt}} = \frac{I_{x}}{D_{x}} + \frac{I_{y}}{D_{y}} + \frac{I_{t}}{D_{t}} \end{aligned}$$

This allows us to define $\mathbf{\psi }$ as

10 $$\begin{aligned} \mathbf{\psi } = g(\sigma ^{2},\tau ^{2}) * \left( \begin{array}{cccccccc} I_{x}I_{x} &{} &{} I_{x}I_{y} &{}&{} I_{x}I_{t} &{}&{} I_{x}I_{z} \\ I_{x}I_{y} &{} &{} I_{y}I_{y} &{}&{} I_{y}I_{t} &{}&{} I_{y}I_{z} \\ I_{x}I_{t} &{} &{} I_{y}I_{t} &{}&{} I_{t}I_{t} &{}&{} I_{t}I_{z} \\ I_{x}I_{z} &{} &{} I_{y}I_{z} &{}&{} I_{t}I_{z} &{}&{} I_{z}I_{z} \\ \end{array} \right) , \end{aligned}$$

where $g(\sigma ^{2},\tau ^{2})$ is a Gaussian smoothing function, with spatial and temporal scales defined by $\sigma$ and $\tau$ respectively.

The set of 4D Harris interest points  is defined as the set of spatio-temporal locations within the sequence, for which *H* is greater than the threshold 
11In Willems et al. (2008), Willems et al. extended the Beaudet Saliency Measure (Beaudet 1978) into the spatio-temporal domain. Rather than the second-moment-matrix of Laptev et al. they calculated the Hessian ($\mu$) of the Gaussian smoothed spatio-temporal volume (*I*). The detected interest points relate to areas with strong second order intensity derivatives, including both blobs and saddles.

As in the 4D Harris scheme, gradients along *z* are estimated using the relationships between the depth and intensity stream gradients. This allows the 4D Hessian $\mu$ to be calculated as

12 $$\begin{aligned} \mu = g(\sigma ^{2},\tau ^{2}) * \left( \begin{array}{cccc} I_{xx} &{}\quad I_{xy} &{}\quad I_{xt} &{}\quad I_{xz} \\ I_{xy} &{}\quad I_{yy} &{}\quad I_{yt} &{}\quad I_{yz} \\ I_{xt} &{}\quad I_{yt} &{}\quad I_{tt} &{}\quad I_{tz} \\ I_{xz} &{}\quad I_{yz} &{}\quad I_{tz} &{}\quad I_{zz} \\ \end{array} \right) . \end{aligned}$$

The set of interest points  is calculated as the set of spatio-temporal locations, for which the determinant of $\mu$ is greater than the threshold 
13


### Interest Points in 3.5D

In part, the Harris and Hessian interest point operators are motivated by the idea that object boundary points are highly salient, and that intensity gradients relate to boundaries. However, depth data directly provides boundary information, rendering the estimation of the intensity gradient along *z* somewhat redundant. An alternative approach would be to employ a “3.5D” representation, using a pair of complimentary 3D spatio-temporal volumes, from the appearance and depth sequences. This can be applied to the Harris measure,14and the Hessian measure15where $\theta$ and $\phi$ are Eq. 8 applied to the appearance and depth streams respectively, while $\xi$ and $\zeta$ are the 3 by 3 Hessians. The relative weighting of the appearance and depth information is controlled by $\alpha$. This approach exploits complimentary information between the streams, to detect interest points where there are large intensity changes and/or large depth changes.

### 3.5D Separable Filters

A third highly successful approach to interest point detection, is the Separable Linear Filters technique of (Dollar et al. 2005). Peaks are detected within a spatio-temporal volume, after filtering with a 2D Gaussian in the spatial dimensions, and a quadrature pair of Gabor filters $h_{ev}$ and $h_{od}$ along the temporal dimension,

16 $$\begin{aligned} S(I) = \left( I * g\left( \sigma ^{2}\right) * h_{ev}\right) ^2 + \left( I * g\left( \sigma ^{2}\right) * h_{od}\right) ^2. \end{aligned}$$

Employing the same 3.5D approach used for the Harris and Hessian detectors, leads to17where *I* and *D* are the appearance and depth streams respectively.

### Dense Trajectories

An alternative scheme to detecting interest points which we explore is accumulation via densely sampled trajectories. In this approach, feature points are densely sampled in the first frame, and are tracked over time by median filtering of the motion field (Wang et al. 2011). Any trajectories which are static are assumed to be part of the background and are ignored. To prevent drift during tracking, an upper limit is placed on the length of the trajectories, after which a new grid of dense points is sampled.

Local features can then be accumulated over the trajectory to form a single descriptor, encompassing the spatio-temporal behaviour of a particular part of the scene.

## Feature Descriptors

Once feature points have been detected, the next stage is to extract descriptors to encode the characteristics of these salient regions for classification. The descriptors can be based on various types of information, including appearance, motion and saliency, but we wish to also include our additional depth information.

### RMD

The Relative Motion Descriptor (RMD) introduced by Oshin et al. (2011) has been shown to perform well in a large range of action recognition datasets, while making use of only the saliency information obtained during interest point detection. A spatio-temporal volume $\eta$ is created, containing the interest point detections and their strengths. The saliency content of a sub-cuboid, with origin at (*u*, *v*, *w*) is defined for a sub-cuboid of dimensions $(\hat{u},\hat{v},\hat{w})$ as

18 $$\begin{aligned} c(u,v,w) = \displaystyle \sum _{{\varvec{\gamma }}=0}^{(\hat{u},\hat{v},\hat{w})} \eta (\left[ u,v,w\right] +{\varvec{\gamma }}). \end{aligned}$$

For efficiency this is implemented as an integral volume. The descriptor $\delta$ of the saliency distribution at a position (*u*, *v*, *w*) can then be formed, by performing *N* comparisons of the content of two randomly offset spatio-temporal sub-cuboids, with origins at $(u,v,w)+{\varvec{\beta }}$ and $(u,v,w)+{\varvec{\beta }'}$:

19 $$\begin{aligned} \delta (u,\!v,\!w) = \displaystyle \sum _{n=0}^{N} \!\!\left\{ \begin{array}{ll} 2^{n}&{} \text {if }c(\left[ u,v,w\right] + {\varvec{\beta }}_{\!n}) \!>\! c(\left[ u,v,w\right] + {\varvec{\beta }'}_{\!\!n}) \\ 0 &{} \!\!\text{ otherwise } \\ \end{array} \right. \end{aligned}$$

Note that the collections of offsets ${\varvec{\beta }}_{0..N}$ and ${\varvec{\beta }'}_{0..N}$ are randomly selected prior to training, and then maintained, rather than selecting new offsets for each clip.

By extracting $\delta$ at every location in the sequence, a histogram may be constructed, which encodes the occurrences of relative saliency distributions within the sequence, without requiring appearance data or motion estimation. Increasing the number of comparisons *N* leads to improved descriptiveness, however the resulting histograms also become more sparse. A common alternative is to compute several $\delta$ histograms, each using different collections of random offsets ${\varvec{\beta }}_{0..N}$ and ${\varvec{\beta }'}_{0..N}$. The resulting histograms are then concatenated, with the result encoding more information without sparsifying the histogram. However, this comes at the cost of the independence between bins, i.e. introducing some possible redundancies.

We propose extending the standard RMD described above, by storing the saliency measurements within a 4D integral hyper-volume, so as to encode the behaviour of the interest point distribution across the 3D scene, rather than within the image plane. The 4D integral volume can be populated by extracting the depth measurements at each detected interest point. RMD-4D descriptors can then be extracted, using comparisons between pairs of sub-hypercuboids. The resulting histogram encodes relative distributions of saliency, both temporally, and in terms of 3D spatial location. As with the original RMD, the descriptor can be applied in conjunction with any interest point detector and is not restricted to the extended interest point detectors described in Section 7 (provided that a depth video is available during descriptor extraction).

### Bag of Visual Words

One of the most successful approaches in action recognition is to concatenate a range of local descriptors and to calculate a bag of words representation. Laptev et al. (2008) used this approach to great effect to combine HOG and HOF descriptors (defined as *G* and *F*). Both histograms are computed over a small window, storing coarsely quantized image gradient and optical flow vectors, respectively. This provides a descriptor $\rho$ of the visual appearance and local motion around the salient point at *I*(*u*, *v*, *w*).

20 $$\begin{aligned} \rho (u,v,w) = \left( G\left( I\left( u,v,w\right) \right) ,F\left( I\left( u,v,w\right) \right) \right) \end{aligned}$$

When accumulating $\rho$ over space and time, a Bag of Words (BOW) approach is employed. Clustering is performed on all $\rho$ obtained during training, creating a codebook of distinctive descriptors. During recognition, all newly extracted descriptors are assigned to the nearest cluster center from the codebook, and the frequency of each clusters occurrences are accumulated. In this work K-Means clustering is used, with a Euclidean distance function as in Laptev et al. (2008). To extend $\rho$ to 4D, we include a Histogram of Oriented Depth Gradients (HODG):

21 $$\begin{aligned} \rho (\!u,\!v,\!w\!)\! = \!\Big (\!G\!\big (I\!\left( \!u,\!v,\!w\!\right) \big )\!,\!F\!\big (I\!\left( \!u,\!v,\!w\!\right) \big )\!,\!G\!\big (D\!\left( \!u,\!v,\!w\!\right) \big )\Big ). \end{aligned}$$

Thus the descriptor encapsulates local structural information, in addition to local appearance and motion. The bag of words approach is applied to this extended descriptor, as in the original scheme. Importantly, this descriptor is not dependent on the interest point detector, provided the HODG can be calculated from the depth stream *D*. By normalising these local descriptors, we are able to resolve the scale ambiguity which remained in our auto-calibration of Sect. 5.

### 3D Motion Descriptors

The inclusion of structural (depth) features into the bag of words descriptor does not fully exploit the additional information in the Hollywood 3D dataset. During pre-processing we also extracted the 3D motion fields for the dataset, which can be used as a replacement for the optical flow features *F*. We refer to these 3D motion descriptors as “Histograms of Oriented Scene-flows” (HOS).

Given the dense 3D flow field ($\dot{x},\,\dot{y},\,\dot{z}$), we can extract a local 3D motion descriptor using the spherical co-ordinate system

22 $$\begin{aligned} {\gamma } = \text {atan}\left( \frac{\dot{y}}{\dot{x}} \right) \text { and } {\delta } = \text {atan}\left( \frac{\dot{z}}{\dot{y}} \right) , \end{aligned}$$

to describe the 3D orientation of flow vectors. Note that ${\gamma }$ refers to the “in plane” orientation (from the viewpoint of the left camera) *i.e*.when ${\gamma }$ is $0^{\circ }$, the motion is toward to the top of the image, when ${\gamma }$ is $90^{\circ }$ the motion is toward the right of the image, etc. In contrast ${\delta }$ refers to the “out of plane” orientation, *i.e*.how much the motion is angled away from, or towards, the camera.

**Fig. 6:**
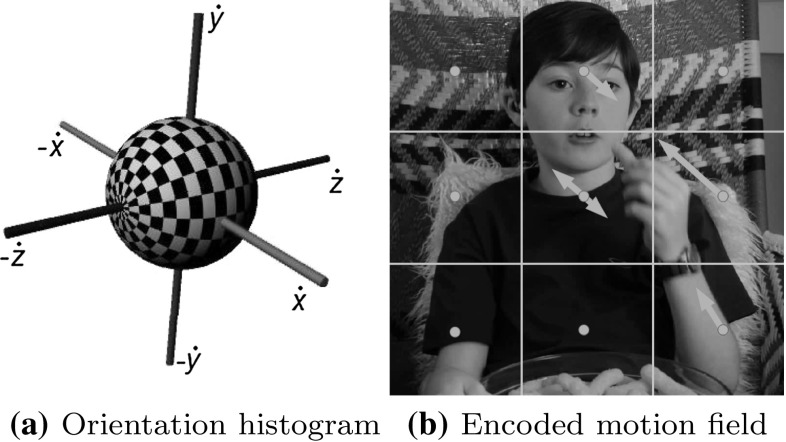
**a** Orientation bins visualised with alternating *white* and *black squares*. ${\gamma }$ is rotation around the *w* *axis*. ${\delta }$ is rotation around the *u* *axis*. **b** a scene divided into a 3 by 3 grid of subregions, with the motion of each subregion aggregated (Color figure online)

We encode the distribution of 3D orientations in a region around each interest point, capturing the nature of local motion field using a spherical histogram $\mathbf{H}$ (see Fig. 6) which can be included into the bag of words descriptor $\rho$. This is similar to the approach used for shape context (Belongie et al. 2002), but in the velocity domain. The contribution of each flow vector to the histogram is weighted based on the magnitude of the flow vector. As with HoG, HoF and HoDG this histogramming discards much of the spatial information. However, some general attributes are maintained by separating the region into several neighbouring blocks, and encoding each of them independently as $\mathbf{H}_{1\ldots n}$. These sub-region spherical histograms are then combined to form the overall descriptor $\mathbf{H}$. It should be noted that placing histogram bins at regular angular intervals in this way leads to the bins covering unequal areas of the sphere’s surface. An exaggerated version of this effect can be seen in Fig. 6a, although in practice fewer bins are used and the difference is less pronounced. In the future, regular or semi-regular sphere tessellations could be considered to mitigate this (Saff and Kuijlaars 1997).

As above we normalise the descriptors to resolve the scale ambiguity between sequences. Thus, even though motion fields are consistent only up to a similarity transform, the normalised spherical histograms,

23 $$\begin{aligned} {\bar{\mathbf{H}}} = \frac{ \mathbf{H}}{| \mathbf{H}|}\,, \end{aligned}$$

are consistent up to a 3D rotation, making these 3D motion descriptors much more comparable between camera configurations, and thus suitable for “in the wild” recognition. In addition to this, the normalised features provide invariance to the speed at which actions are performed, as only the shape and not the value of the motion field is encoded. This is again very important for “in the wild” recognition, with many different actors, each of whom have their own action style.

### Rotational Invariance

Next we look at including viewpoint invariance in our 3D motion features (i.e. removing the final 3D rotation ambiguity, and making the descriptors completely consistent). This is one of the biggest challenges for “in the wild” action recognition. The the same action viewed from different angles looks completely different. However, as we are using the underlying 3D motion field, it is possible to modify our feature encoding to be invariant to such changes.

**Fig. 7:**
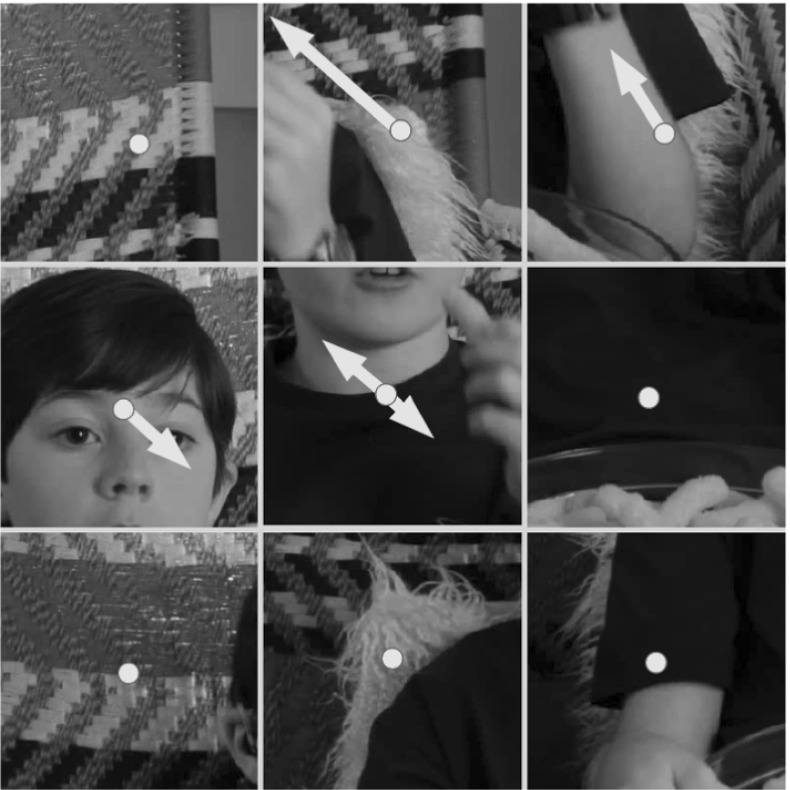
${\bar{\mathbf{H}}^{\mathbf{r}}}$ The subregions of the encoded motion field are re-arranged such that the region of maximum motion occurs first. This provides some degree of invariance to camera roll

We firstly encode invariance to camera roll (i.e. rotation around the z axis) by cycling the order of the subregion histograms $\mathbf{H}_{1\ldots n}$ such that the subregion containing the largest amount of motion occurs first [similar to the orientation normalisation used in shape context (Belongie et al. 2002), SIFT (Lowe 2004), Uniform LBPs (Ojala et al. 2002) etc.]. This re-arranged, roll-invariant, descriptor is referred to as ${\bar{\mathbf{H}}^{\mathbf{r}}}$ (see Fig. 7).

We can follow a similar approach for the flow vectors within the subregion histograms, to make the direction of the motions as well as their positions, rotationally-invariant. If we find the strongest motion vector in $\mathbf{H}$ and label its 3D orientation as $\hat{\phi }$,$\hat{\psi }$ then we can redefine our local orientations in relation to this flow vector,

24 $$\begin{aligned} {\gamma } ^p= \text {atan}\left( \frac{\dot{y}}{\dot{x}}- \hat{\phi } \right) \text { and } {\delta } ^p = \text {atan}\left( \frac{\dot{z}}{\dot{y}}- \hat{\psi } \right) . \end{aligned}$$

**Fig. 8:**
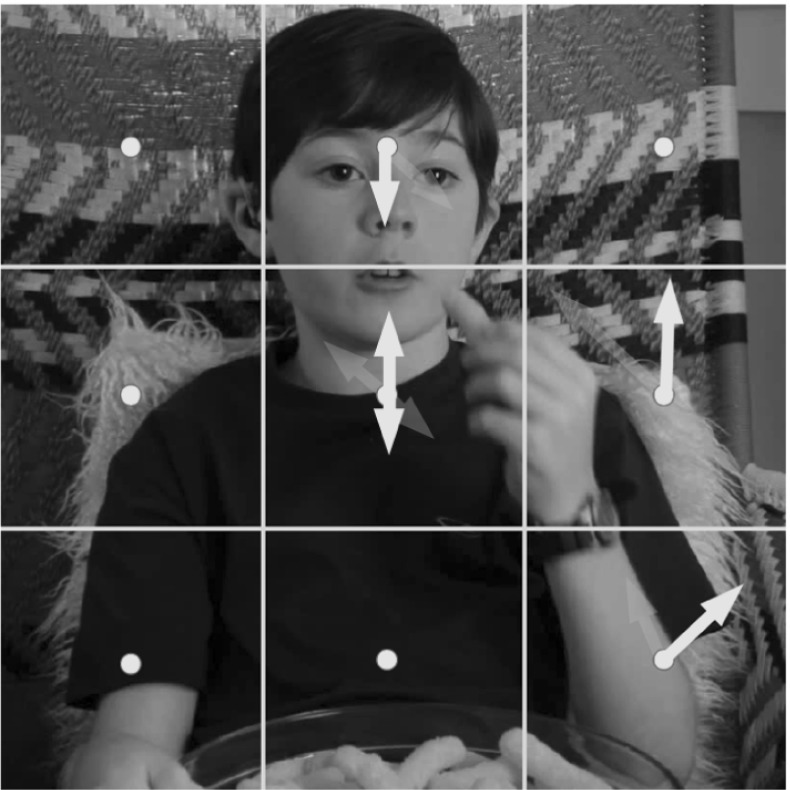
${\bar{\mathbf{H}}^{\mathbf{p}}}$ The orientation of the strongest motion vector in the scene is used to normalise the histograms, providing robustness to camera pitch and roll

The resulting descriptors ${\bar{\mathbf{H}}^{\mathbf{p}}}$ obtained when encoding ${\gamma } ^p$,${\delta } ^p$ makes the flow vectors robust to camera pitch (rotation around the x axis) in addition to roll, as shown in Fig. 8.

**Fig. 9:**
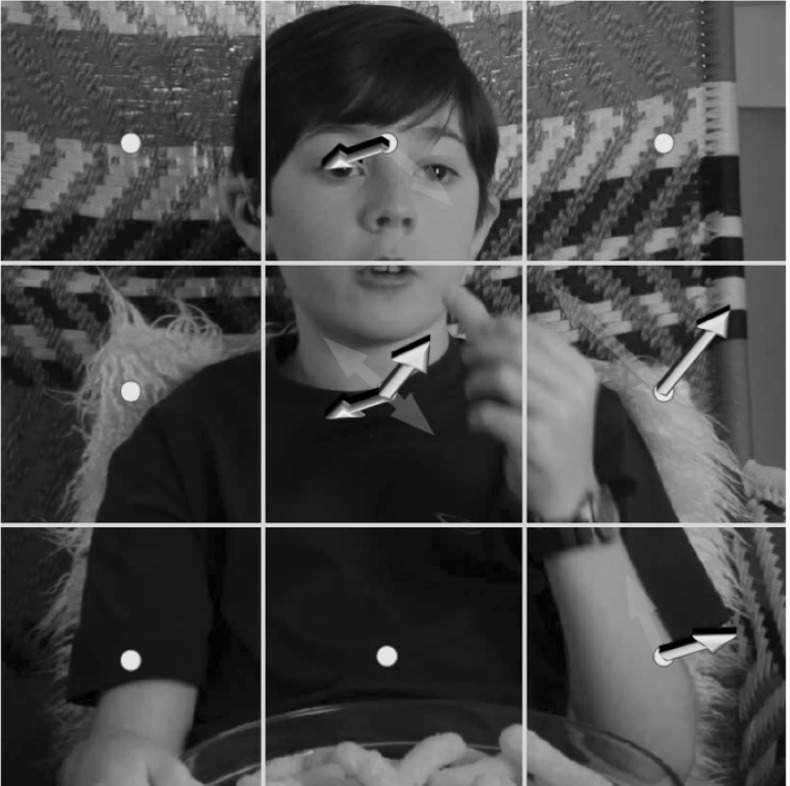
${\bar{\mathbf{H}}'}$ A new set of 3D axes is chosen using PCA, relating to the dominant 3D motion orientations in the scene. This provides complete invariance to camera viewpoint change

However, due to the separation of ${\gamma }$ and ${\delta }$ our descriptors are still not resistant to camera pans (rotation around the y axis, which at 90$^{\circ }$ causes ${\gamma }$ orientation to become ${\delta }$ orientation). In addition, normalising based on the maximum flow vector is sensitive to outliers in the flow field. As such, our final approach is to perform PCA on the local region of the motion field, extracting 3 new basis vectors $\dot{x}',\dot{y}',\dot{z}'$ (the eigenvectors of the motion field covariance). Computing orientation using these basis vectors,

25 $$\begin{aligned} {\gamma } '= \text {atan}\left( \frac{\dot{y}'}{\dot{x}'} \right) \text { and } {\delta } ' = \text {atan}\left( \frac{\dot{z}'}{\dot{y}'} \right) , \end{aligned}$$

leads to a descriptor ${\bar{\mathbf{H}}'}$ which is invariant to all 3 types of camera viewpoint change, and also robust to outlier motions. See Fig. 9 for an illustration.

## Results

Classification was performed for all tests, with a multi-class SVM using an RBF kernel. Note that tests were performed using other SVM kernels including linear, $\chi ^{2}$ and multi-$\chi ^{2}$. However, linear kernels were found to perform poorly while $\chi ^{2}$ kernels greatly increased computation time and had little effect on performance. Thus for clarity we only present the results using RBF kernels. To facilitate comparisons with the Hollywood 1 and 2 datasets, the Average Precision (AP) measure was used, as explained in the PASCAL VOC (Everingham et al. 2010). Relevant source code is available online along with the data (Hadfield and Bowden). RMD tests were performed with 4 binary comparisons per histogram ($N=4$), concatenating 10 descriptor histograms. BOW tests were performed with 4,000 cluster centres (as suggested in Laptev et al. 2008), with the local histogram descriptors calculated using a block size of 3 by 3, with 8 orientation bins. For the 3D motion (HOS) features, each subregion histogram uses $4 \times 4$ bins in the ${\gamma }$ and ${\delta }$ orientations.

To aid clarity, we will first examine 3 components of the framework independently; the interest point detection, the local descriptors, and the motion feature encoding. We will then summarise the most effective technique in each area and compare against other techniques designed for “in the wild” 3D action recognition, including (Konda and Memisevic 2013) which uses auto-encoders to implicitly model uncalibrated structural information, and (Iosifidis et al. 2014a, b) which use Extreme Learning Machines on Dense Trajectory encoded HoG/HoF/MBH descriptors.

### Interest Point Analysis

First we examine the benefits of including depth information during interest point detection. Clearly this is the least significant stage of the pipeline to include depth; no information from the depth stream is encoded in the descriptors, and depth information cannot be used by the classifier to distinguish actions. Instead, the depth stream is used only to make more informed decisions about which regions of the appearance stream to encode and which to discard. This is particularly true after the encoded regions are accumulated into a single holistic descriptor.

For these comparisons the standard Bag of Words descriptor (see Sect. 8.2, HoG/HoF/HoDG) is used. The traditional spatio-temporal interest points (Separable Filters 3D-S and Harris Corners 3D-Ha) are compared to the proposed depth aware interest point detectors and the currently state-of-the-art Dense Trajectories approach (also using the 3D Bag of Words descriptor). The AP for each class is shown in Table 2, with bold entries indicating performance greater than both of the standard spatio-temporal schemes.

**Table 2:** Average precision per class, on the 3D action dataset, for a range of sparse interest point detectors, including simple spatio-temporal interest points, depth aware extensions and Dense Trajectory encoding

Action	3D-S	3D-Ha	**4D-He**	**4D-Ha**	**3.5D-S**	**3.5D-He**	**3.5D-Ha**	Dense-Traj
*NoAction*	11.4	12.1	**12**.**2**	**12**.**9**	11.4	12.0	**13**.**7**	**14**.**2**
*Run*	12.6	19.0	15.9	**22**.**4**	12.7	**21**.**8**	**27**.**0**	**27**.**3**
*Punch*	2.9	10.4	2.9	4.8	2.9	5.7	5.7	9.4
*Kick*	3.6	9.3	4.2	4.3	3.8	3.7	4.8	**9**.**9**
*Shoot*	16.2	27.9	18.9	17.2	16.2	16.2	16.6	**33**.**8**
*Eat*	3.6	5.0	3.6	**5**.**3**	3.6	**7**.**7**	**5**.**6**	**11**.**9**
*Drive*	15.3	24.8	**25**.**6**	**69**.**3**	15.5	**76**.**5**	**69**.**6**	**56**.**8**
*UsePhone*	6.5	6.8	**14**.**7**	**8**.**0**	6.5	**17**.**7**	**7**.**6**	**12**.**4**
*Kiss*	6.5	8.4	**8**.**5**	**10**.**0**	6.5	**9**.**4**	**10**.**2**	**11**.**4**
*Hug*	2.6	4.3	3.5	**4**.**4**	2.6	3.4	**12**.**1**	5.3
*StandUp*	6.8	10.1	7.0	7.6	6.9	9.1	9.0	**17**.**6**
*SitDown*	4.2	5.3	4.5	4.2	4.2	4.3	**5**.**6**	**6**.**7**
*Swim*	5.5	11.3	7.8	5.5	5.5	5.9	7.5	8.4
*Dance*	2.3	10.1	4.2	**10**.**5**	2.2	3.8	7.5	**26**.**5**
Average	7.1	12.6	9.8	**13**.**3**	7.1	**13**.**4**	**14**.**1**	**17**.**9**

The Bag of Visual Words (HoG/HoF/HoDG) feature encoding was used. Classes are shown in bold, for schemes outperforming both of the simple spatio-temporal interest point schemes

The type of saliency measure used has a surprisingly large effect on the performance, with the average performance for the best scheme being more than double that of the worst, even using the same feature encoding. For the standard spatio-temporal schemes, Harris points (3D-Ha) outperform separable filter points (3D-S) for all actions. This is also reflected in the depth aware schemes, and is unsurprising, as separable filters were designed primarily for computational speed. Hessian based interest points prove less informative than the extended Harris operators in both the 4D and 3.5D case. For all detectors, the 4D schemes outperform their standard spatio-temporal counterpart, while the 3.5D approach proves more informative than the direct 4D extension. This confirms the belief that the calculation of intensity gradients along *z* is redundant, and that the combination of intensity and structure gradients is a stronger measure of saliency. The Dense-Trajectory approach proves to be the more descriptive than the sparse interest points, when used to encode the same feature descriptors. However this comes at the price of significantly increased computational cost due to it’s dense nature.

Interestingly, certain actions consistently perform better, when described by depth aware interest points. These are actions such as *Kiss*, *Hug*, *Drive* and *Run* where there is an informative foreground object, which depth aware interest points are better able to pick out. In contrast, actions such as *Swim*, *Dance* and *Shoot* are often performed against a similar depth background, or within a group of people, and the inclusion of depth in the saliency measure is less valuable. This suggests that a combination of standard spatio-temporal, and depth aware schemes may prove valuable.

The complexity of the depth aware interest point detectors remains of the same order as their spatio-temporal counterparts (linear with respect to *u*, *v* and *w*). Naturally the multiplicative factor is increased however, with 3.5D techniques being roughly twice as costly, and 4D techniques taking 4 times as long.

### Structural Descriptor Analysis

Next, the use of structural information at the feature level was explored, using the best performing Interest Point detection schemes from the previous analysis. These results are shown in Table 3. The best performing descriptor for each saliency measure is shown in bold. The RMD descriptor has it’s own holistic accumulation scheme and so does not fit well with Dense Trajectory encoding. However, the Bag of words descriptor can be evaluated in both sparse and dense scenarios.

**Table 3:** Correct classification rate and average precision for different local features using the 2 top performing saliency measures

Descriptor	Saliency	CC	AP
RMD	3.5D-Ha	12.3	11.9
RMD-4D	3.5D-Ha	17.2	14.4
HoG/Hof	3.5D-Ha	17.9	13.0
HoG/Hof/HoDG	3.5D-Ha	**21**.**8**	14.1
HoG/Hof/HoDG	Dense-Traj	20.8	**17**.**9**

The best feature for each saliency measure is shown in bold

It should be noted that previous work using Dense Trajectories has employed them as both an accumulation scheme, and as a feature descriptor. It is possible that this could provide additional information, further improving performance. However, the purpose of this analysis is to quantify the value of depth based features. Further, it should be noted that features such as Eigenjoints (Yang et al. 2012) or Actionlets (Wang et al. 2012) (which are currently state-of-the-art for non-“in the wild” 3D action recognition) cannot be evaluated as the user masks and body skeletons normally provided by the Kinect, cannot be produced in this more complicated scenario.

Both types of descriptor show a consistent improvement when incorporating structural information, with increases of around 20 % in both average precision and correct classification. This demonstrates the value of such features for recognizing actions in the wild. Overall, the Bag of Words descriptors perform somewhat better than the RMD descriptors. This is unsurprising as the RMD relies only on interest point detections, without the inclusion of any visual and motion information.

The complexity of the RMD-4D is greater than the standard RMD (being linear in the range of depth values, as well as in *u*, *v* and *w*). This is somewhat mitigated by the use of integral volumes however, meaning that runtimes are still on the order of seconds using a single CPU. In contrast the extraction of HoDG features relates to only a 50 % increase in runtime of the standard bag of words descriptor (although the complexity remains linear). However the increased feature vector length does lead to an increased cost during codebook generation, as K-Means is generally linear in the number of dimensions.

### Motion Descriptor Analysis

Taking the best descriptor so far (HoG/HoF/HoDG), we next investigate improvements to the motion based portion of the descriptor, in light of the available depth information. This set of experiments was performed using the 3.5D-Ha interest point detector.

**Table 4:** Per class average precision scores using various types of motion features, including 2D motions, uncalibrated 3D motions, unnormalised 3D motions, and calibrated motions encoding varying levels of invariance to camera viewpoint change

Action	HOF	${\bar{\mathbf{H}}}$-uncal	${\bar{\mathbf{H}}}$	$\mathbf{H}$	${\bar{\mathbf{H}}^{\mathbf{r}}}$	${\bar{\mathbf{H}}^{\mathbf{p}}}$	${\bar{\mathbf{H}}'}$
*NoAction*	12.5	13.0	18.0	16.2	17.2	15.3	**21**.**2**
*Run*	18.0	21.5	44.3	41.1	40.8	55.9	**63**.**1**
*Punch*	2.9	10.9	48.7	45.6	51.6	52.1	**54**.**2**
*Kick*	3.6	8.1	18.2	18.2	**19**.**9**	18.1	**19**.**9**
*Shoot*	16.3	24.4	27.1	26.5	30.2	27.9	**31**.**0**
*Eat*	3.6	5.5	24.2	24.1	24.0	23.1	**24**.**2**
*Drive*	35.1	45.4	**62**.**3**	58.4	62.0	50.2	60.8
*UsePhone*	8.1	7.8	18.8	18.2	19.3	18.2	**22**.**3**
*Kiss*	6.7	7.0	24.2	24.1	24.0	26.3	**31**.**3**
*Hug*	2.6	3.5	21.8	21.0	22.2	23.8	**32**.**4**
*StandUp*	8.8	7.1	49.1	47.0	**51**.**8**	49.0	50.0
*SitDown*	4.3	4.8	16.3	14.1	17.9	16.9	**18**.**1**
*Swim*	6.4	14.0	28.8	27.1	30.0	**43**.**2**	43.0
*Dance*	2.8	3.7	45.3	41.8	44.2	**48**.**1**	44.9
Overall	9.4	12.6	31.9	30.2	32.5	33.4	**36**.**9**

Classes are shown in bold, for schemes outperforming the 2D motion features

**Table 5:** The current state of the art for in the wild 3D action recognition

Algorithm	SAE-MD	MVRELM	Disp-Pyr	Enriched
	(Av)(Konda and Memisevic 2013)	(Iosifidis et al. 2014b)	{1,3}(Iosifidis et al. 2014a)	IPs(Mademlis et al. 2014)
mAP	26.1	29.9	30.5	30.1

Algorithm	3.5D	Structure	3D Motion	Den-Traj HOS
	IPs	Features	(HOS’)	HoG/HoDG
mAP	14.1 (+12%)	17.9 ($+$22 %)	36.9 ($+$293 %)	37.4

For each of our depth-aware extensions, the improvement over spatio-temporal techniques is shown in parentheses

In Table 4 we can see that the raw 3D motion features (${\bar{\mathbf{H}}}$-uncal), directly attainable from the dataset with a generic calibration, perform rather poorly, offering only a minor improvement over 2D motion based features (*HOF*Hadfield and Bowden (2013)). The use of our proposed stereo sequence auto-calibration (${\bar{\mathbf{H}}}$) dramatically improves performance, almost tripling the average precision, by removing the projective distortion effects on the motion field. This helps to explain why 3D motion estimation techniques have not previously been exploited for “in the wild” action recognition, despite the fact that actions are generally defined by their 3D motions. The results also show that the unnormalised features ($\mathbf{H}$), which are not scale invariant, perform uniformly worse than their normalised counterparts. It is worth noting, however, that Hollywood 3D does not contain the *Run*/*Jog*/*Walk* ambiguities of some datasets. Instead the wide range of viewpoints and zooms present in the data favour the more consistent ${\bar{\mathbf{H}}}$ features.

The viewpoint invariant encoding schemes of Sect. 8.4 (upgrading the motion fields to fully consistent, rather than “up to a rotation”) provide more modest improvements. Including roll invariance (${\bar{\mathbf{H}}^{\mathbf{r}}}$) gives only a small performance increase, probably because broadcast footage such as that contained in the Hollywood-3D dataset contains few camera rolls. It may be expected that this scheme would prove more valuable in other scenarios such as on mobile devices. Attempting to include pitch invariance (${\bar{\mathbf{H}}^{\mathbf{p}}}$) by normalising motion orientations actually reduces performance on many of the action classes. This is likely because normalising by the maximum motion makes the technique susceptible to outliers in the motion field. It is interesting to note however, that there is a marked improvement for a small number of actions such as *Run* and *Swim*. This may be because these actions experience greater variation in camera pitch (for example running shots being seen from above, and swimming shots from underwater). In addition, the motions are generally stronger for these actions which may make the dominant direction more reliable. The final scheme (${\bar{\mathbf{H}}'}$), including full viewpoint invariance by estimating new motion orientation axes, provides the best performance. It is interesting to note that all of these encoding schemes actually discard some of the information present within the original features. However, for the task of “in the wild” action recognition, camera viewpoint invariance outweighs this, by making it easier to generalise between sequences.

It should be noted that there are more advanced features such as Motion Boundary Histograms (MBH) which have proven very powerful for 2D action recognition in recent years. However, depth-aware extensions of these complex features are beyond the scope of this work.

### Current State of the Art

From this extensive analysis, our best approach to “in the wild” 3D action recognition is to use the Dense Trajectory encoding scheme, combined with the bag-of-words descriptors including 3D structure and motion. In addition, we found that calibrated 3D motion features are far more powerful than their 2D counterparts, especially when encoded with full viewpoint invariance (${\bar{\mathbf{H}}'}$). In Table 5 we show these 3 techniques independently (without using depth aware components in the rest of the pipeline) against the combination of all 3 techniques in a single framework. We also show results for the other techniques currently submitted to our online leaderboard [13], including several deep-learning based techniques. Clearly the calibrated 3D motion features (HOS) offer the largest improvements, but all of the proposed techniques offer significant improvement over their spatiotemporal counterparts (quantified in parentheses). In addition these gains are complementary, and in combination provide a more than three-fold improvement in performance.

## Conclusion

In this paper, we introduced a large publicly available corpus of 3D data (and code) for the action recognition community to compare techniques in natural environments. Further, we have demonstrated the intrinsic value of this 3D information throughout the Natural Action Recognition pipeline. Specifically, a variety of new interest point detection algorithms incorporating depth data have been shown to improve action recognition rates, doubling performance in some cases, even using standard features. Additionally, popular feature descriptors have been modified to encode structural information, demonstrating an average of 20 % additional improvement in performance. We have also discussed the use of 3D information for estimating a new, more advanced class of motion features based on scene-flow. These provide recognition rates significantly better than previously state-of-the-art techniques, particularly when utilising the proposed viewpoint-invariant encoding.

In fact, our results demonstrate that invariances are vital for features used in recognition “in the wild”. The proposed robust stereo sequence calibration step is needed to fully exploit the power of 3D information in the presence of large intra-class variation. As a result, the estimated calibrations for the dataset have been made publicly available, in addition to the stereo data, reconstructed depth and code for the baseline techniques.

Future work should focus on more complex feature descriptors, particularly focused on mitigating the sparsity which may arise in higher dimensional feature spaces. It would also be useful to develop further the invariant holistic encoding schemes for local features, preserving the invariances encoded in our 3D motion features without discarding so much relational information.

## Footnotes

1 Drive Angry, Tron: Legacy, My Bloody Valentine, Spy Kids: All The Time In The World, A Very Harold and Kumar Christmas, Final Destination 5, Underworld: Awakening, Step Up 3D, Sanctum, Avatar, Resident Evil: Afterlife, Pirates Of the Caribbean: On Stranger Tides, The Three Musketeers and Fright Night
